# Potential Elucidation of a Novel CTL Epitope in HIV-1 Protease by the Protease Inhibitor Resistance Mutation L90M

**DOI:** 10.1371/journal.pone.0071888

**Published:** 2013-08-28

**Authors:** Werner Smidt

**Affiliations:** Bioinformatics and Computational Biology Unit, Department of Biochemistry, University of Pretoria, Pretoria, South Africa; Institut Pasteur, France

## Abstract

The combination of host immune responses and use of antiretrovirals facilitate partial control of human immunodeficiency virus type 1 (HIV-1) infection and result in delayed progression to Acquired Immunodeficiency Syndrome (AIDS). Both treatment and host immunity impose selection pressures on the highly mutable HIV-1 genome resulting in antiretroviral resistance and immune escape. Researchers have shown that antiretroviral resistance mutations can shape cytotoxic T-lymphocyte immunity by altering the epitope repertoire of HIV infected cells. Here it was discovered that an important antiretroviral resistance mutation, L90M in HIV protease, occurs at lower frequencies in hosts that harbor the B*15, B*48 or A*32 human leukocyte antigen subtypes. A likely reason is the elucidation of novel epitopes by L90M. NetMHCPan predictions reveal increased affinity of the peptide spanning the HIV protease region, PR 89–97 and PR 90–99 to HLA-B*15/B*48 and HLA-A*32 respectively due to the L90M substitution. The higher affinity could increase the chance of the epitope being presented and recognized by Cytotoxic T-lymphocytes and perhaps provide additional immunological pressures in the presence of antiretroviral attenuating mutations. This evidence supports the notion that knowledge of HLA allotypes in HIV infected individuals could augment antiretroviral treatment by the elucidation of epitopes due to antiretroviral resistance mutations in HIV protease.

## Introduction

Infection by Human Immunodeficiency Virus-1 (HIV-1) ultimately leads to the debilitating condition known as Acquired Immunodeficiency Syndrome (AIDS) [1]. Infection is hallmarked by a steady decline in the T-helper cells and failure of adaptive immunity leading to an increased susceptibility to opportunistic infections. Initial immune responses are effective in the control HIV-1 infection. An important defense against HIV-1 infection, is the destruction via apoptosis of infected T-helper cells and has been shown as a major contributor to the control of viremia [2]. The effectors of the response, Cytotoxic T-lymphocytes (CTLs), scrutinize nucleated cells in the body and induce apoptosis in cells that present foreign peptides, usually 9–11 amino acids in length, on the cell membrane by Human Leukocyte Antigen (HLA) class I. Extraction of peptides from a parent protein that is presented by HLA-class I usually involve proteasomal digestion of the parent protein, followed by transport of the resultant fragments into the endoplasmic reticulum where final trimming and loading of the peptide onto the HLA-class I binding groove occurs [3].

Binding to the HLA binding groove is sequence specific and only peptides that have sufficient affinity to an HLA binding groove can be presented. Polymorphisms within the HLA genes coding for the binding pockets have a profound effect on the binding motif and thus the set of presented peptides from HIV-1 protein products can differ between individuals [4]. The peptide sequence also determines the effectivity of CTL responses it induces and therefore different HLA allotypes can provide varying degrees of protection. For instance, HLA subtypes such as HLA-B*58 and HLA-B*27 provide prolonged protection against HIV-1 infection and are associated with delayed onset of AIDS, while the converse is true of HLA-B*08 [5], [6], [7], [8].

Rapid mutations of the HIV-1 genome due to erroneous HIV Reverse Transcriptase (RT) [9], [10] and host RNA processing elements [11], cause immunity escape mutations to accumulate in the HIV-1 genome. CTL escape mutations may interfere with the processing and presentation of the CTL epitope or attenuate CTL T-Cell receptor interaction with the peptide-MHC complex rendering the epitope ineffective [12], [13]. In the majority of HIV-1 infected individuals, effective immune responses are mostly transient mainly due to the acquisition of immunity escape mutations by HIV-1. The development of antiretrovirals have provided additional protection against HIV infection. Antiretroviral drugs, such as HIV protease inhibitors (PI) and HIV reverse transcriptase inhibitors (RTI) inhibit steps in the HIV-1 viral replication cycle and have a large negative impact on viral load [14]. Still, ARV resistance can also accumulate, the mechanism involving interference with the binding of an ARV to the target site and rendering the drug ineffective [15].

Previous researchers have provided evidence of interaction between ARV resistance mutations and CTL escape mutations [16]. For example, the HIV-1 protease (PR) resistance mutation M46I is a CTL escape mutation of an HLA-A*02 restricted epitope, but also serves as a resistance mutation to the PIs, tripanavir and atazanavir [17]. Another PR mutation, L90M, elucidates an HLA-A*02 restricted epitope spanning the PR positions 76–84 by inducing an appropriate proteasomal cleavage site [18]. Indeed, this epitope is immunogenic enough to supplant the Gag immunodominant HLA-A*02 restricted epitope, SLYNTVATL. It has also been shown how a HLA-B*15 restricted CTL epitope, KMIGGIGGF of PR is attenuated by a saquinavir related mutation in drug exposed (DE) patients and is normally not found in HIV sequences obtained from drug naïve (DN) HLA-B*15+ patients [19]. The role of CTL responses is a key factor in persistent low-level viremia in patients undergoing antiretroviral therapy where drug resistance has accumulated [20].

The varying nature of peptide binding motives for the vast amounts of HLA allotypes, makes the experimental detection of HLA allotype specific CTL epitopes a laborious process. With the increase in computational power and availability of data, computational methods applied in immunological studies have become possible [21], [22]. As a consequence, various computational tools predicting scores for steps involving the processing an presentation of an MHC binder have been developed, including proteasomal cleavage, TAP transport and MHC affinity [23], [24], [25]. To the author's knowledge, only two tools exists for predicting the immunogenicty, or the avidity of a potential epitope, namely POPI and POPISK, but are limited to the HLA-A2 serotype [26], [27].

Even though these tools are sometimes limited in their accuracy, largely depending on the amount of data they are constructed with, they can provide insights into the mechanism of the emergence of an epitope or the reason for CTL immunity escape. For instance by predicting a lower affinity of a peptide to HLA class I due to the substitution of a favorable residue in an anchor position for a deleterious residue that attenuates or abrogates the ability of a peptide to bind to the particular HLA allotype. In the case of the HLA-B*44 restricted PR 34–42 epitope EEMNLPGRW, the escape mutation PR D35E is predicted by the MHC binding predictor, NetMHCPan, to have a dramatic negative impact on peptide affinity to HLA–B*4402, possibly diminishing the presentation potential of the epitope on HLA–B*4402 [28].

Using statistical methods to infer mutations significantly associated with CTL immunity escape is a challenging task as most public available sequences are rarely annotated with the patient's HLA genotype. This is especially true of sequences obtained from patients that are not treatment naïve. Fortunately, other researchers have found relationships between amino acid substitutions in HIV-1 proteins and HLA allotypes and can potentially be used to provide HLA annotation from HIV-1 protein sequences [29].

Here, it was discovered that there are possible interactions between a common and important protease inhibitor resistance mutation, L90M, and the HLA subtypes B*15, B*48 and potentially A*32. Using the aforementioned data correlating relating amino acid substitutions with HLA subtype, patients were assigned HLA subtypes and the frequencies of L90M were compared between sequence sets of patients that are HLA B*15/B*48/A*32 positive and those that are not. Furthermore, using computational tools that predict HLA binding affinity and proteasomal cleavage, a potential causative immunological component was discovered that could possibly drive the diminished frequency of L90M in the B*15, B*48 and A*32 subtypes. Two potential epitopes, LMTQIGCTL restricted to HLA subtypes B*15 and B*48 as well as MTQIGCTLNF, restricted to the HLA A*32 subtype were discovered. This research provide support, at least in principle, that the HLA genotype could provide a protective component and that appropriate protease inhibitors for which L90M is a preferred mutation should be considered in patients matching the HLA subtypes B*15, B*48 and A*32.

## Results

In this section, the importance of L90M will be demonstrated by measuring its effect on viral load in patients undergoing antiretroviral therapy. With the sparsely available HLA annotation data, the frequency of L90M in patients with HLA annotations were compared. Next, it was discovered that certain HLA subtypes associated mutations were enriched in sequence sets from drug exposed individuals that are devoid of L90M. From HLA assignment data, the frequency of occurrence of L90M were measured. The influence of antiretroviral choice was explored to determine if bias was introduced resulting in lower observed frequency of L90M. Regions spanning L90M were submitted to NetMHCPan for affinity analysis to HLA allotypes belonging to the HLA B*15, B*48 and A*32 subtypes. Finally, HLA associated enriched within the PR region proximal to L90M and potential immunological escape inferred for the proposed novel epitopes.

### The importance of L90M as a PI resistance mutation

An important PR mutation observed in HIV sequences from drug exposed individuals, is L90M [30], [31], [32], [33]. This mutation, beyond being a major resistance contributor to nelfinavir, is an accessory mutation for all but two of the listed PIs. From the sampled sequences analyzed, it occurs in 42% of patients receiving drug therapy vs 2% of patients that are drug naïve (Fisher exact test, 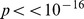). The effect of L90M also has a positive effect on viral load. Comparing viral loads in patients expressing the PR L90M mutation with those that do not, an increased viral load of 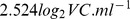 (Mann-Whitney, 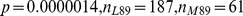, VC  =  Viral copies) was calculated. This was tested under the condition that the PR sequences from which the viral load annotation was obtained, expressed at least three other PI-resistance related mutations and not annotated as drug naïve. It is thought that selection against L90M mutation in HIV treated patients could have a positive effect on treatment outcome.

### Observation of the protease L90M substitution frequencies in sequences annotated with patient HLA data

**Figure 1:**
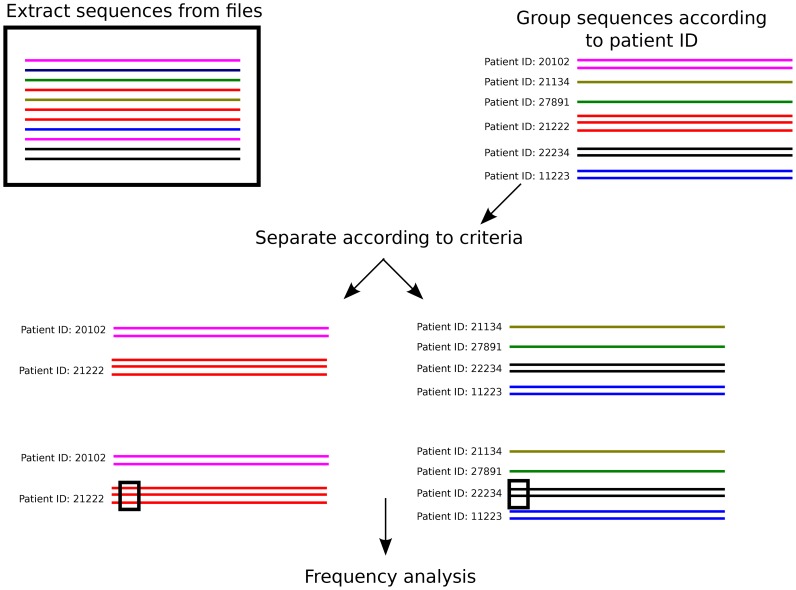
Assignment of sequence to groups according to patient ID and other criteria. This figure demonstrates the process of sequence assignment to the patient ID in the FASTA header line. At first, sequences are divided based on being annotated as drug naïve or non-drug naïve. Subsequently, sequences are assigned to groups based on the patient ID field in the FASTA header. When subsequent criteria are used to further assign groups, it is done in a patient ID fashion, i.e. the patient ID sets from two or more categorized sequence groups are mutually exclusive. The black rectangle over the sequences indicate the procedure of amino acid frequency count. Therefore, the amino acid count of a position specific residue is assigned to the patient as a binary value.

It was tested whether lower frequencies of L90M could be observed in PR sequences obtained from patients that are positive specific HLA subtypes. Here, a sequence set refers to the collection of sequences obtained from various patients. The procedure is highlighted in Figure 1. Due to the varying amount of sequences available for each patient and the bias it could introduce in frequency analysis, a binary value was used to indicate a mutation occurring at least once in PR sequences obtained from each patient. It was thought that low occurrences of a mutation in the sequence set of a patient may not accurately represent the status quo of selective pressures on the HIV genome. However, this may unjustly amplify the residue corresponding to the residue observed in the wild type PR sequence. To mitigate this effect, the residue at a position corresponding to the consensus residue was only counted if it occurred exclusively at that position in the patient's sequence set. For example, considering a patient from which ten PR sequences were obtained and position 46 containing the residues MMMMMMMIIL, the counts would be 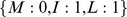. If the residues were MMMMMMMMMM, the counts would be 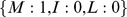. Using this methodology, Fisher exact tests on the occurrence of L90M in PR were performed between patient sequence sets that. Only two allotypes were associated with diminished levels of L90M in PR, namely subtypes A*02 and B*15. negatively with the L90M B*15 and A*02. The results are listed in Table 1. To account for multiple tests, a q-value analysis was performed using the calculated p-values across all positions. The q-value is a measurement of the false discovery rate of a statistic deemed significant [34]. The high q-value calculated for the p-value of B*15 lowers the confidence in this significant result. A likely reason is the small sample size used for the calculation, i.e. sequence data from only 35 patients vs subtype A*02 which had a sample size of 105 patients. The only mitigation factor would've been to increase the amount of sequence from patients with subtype B*15 annotation. However, this data was unavailable.

**Table 1:** Fisher exact test results related to the observed frequencies of L90M.

HLA	90M count (HLA+)	90M count (HLA−)	Fisher exact test p-value	q-value
A*02	2/1032	18/153	0.007	0.070
B*15	0/35	18/153	0.047	0.541
B07	7/90	21/478	0.195	0.930

The results for the Fisher test comparing observed frequencies of L90M in HIV-1 subtype B PR sequences from patients with HLA annotation. The HLA subtype, B07 is included as an example yielding non-significance.

### Lower frequencies of L90M are observed in sequences with assigned HLA subtypes A*32, B*48 and B*15

The possibility was explored to assign putative HLA subtypes to patients using mutation data of PR and HIV-1 reverse transcriptase (RT) regions in Pol sequences. It was first assessed what substitutions are enriched in PR and RT in the absence of L90M. Sequence sets from patients annotated as drug exposed (DE, or non-treatment naïve) were divided into two groups based on the presence of PR L90 or PR M90 respectively in their Pol sequences. Mutations that occur at a significantly higher frequency in the L90 set (Fisher exact test, 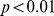) were cross referenced with a list of PR and RT mutations associated with HLA subtypes [18]. The list contains information on position specific residues of HIV-1 and how they relate to to HLA subtype restricted CTL escape, including p-value and q-value statistical measurements. Substitutions conferring escape for CTL epitopes restricted to HLA subtypes A*32, B*07, B*13, B*15, B*44, B*48, B*51, B*52, C*04, C*14 and C*15 were enriched in the PR L90 set. Results of the Fisher exact test measuring substitution frequency differences between the PR L90 and PR M90 sets that are also associated with CTL escape are shown in Table 2. However, there are numerous cases where certain substitutions are associated with both CTL escape and PI resistance [17]. The residues associated HLA subtypes B*13, B*42, B*44, B*51, C*04 and C*15 only occurred in PR and these subtypes were excluded from subsequent analysis. HLA assignment was performed using HLA-associated substitution information for the remaining subtypes A*32, B*07, B*15, B*40, B*48, B*52 and C*15. Mutations suitable for HLA assignment had to meet certain criteria. They had to occur in RT, they needed to be indicative of CTL escape, the q-value needed to be 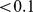 and the frequency of occurrence needed to be 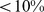. Failure to meet any of the criteria was likely to result in false HLA assignment. An HLA subtype was assigned to a patient if at least one of the HLA subtype restricted CTL escape mutations occurred in RT obtained from them. After HLA assignment, the frequency of L90M for each HLA assigned sequence set was measured. With the additional substitutions included in HLA assignment, it was discovered that only subtypes A*32, B*15 and B*48 still showed significantly lower frequences of L90M.

**Table 2:** Significant mutation frequency differences in PR and RT between sequences with PR L90 and PR M90.

Protein	Mutation	L90	M90	Odds Ratio *log_2_*	p-value	q-value	HLA-Subtype/State/Direct
PR	A71V	258/1127	552/864	−2.57	<<2×10^−16^	<<0.001	B*15/Adapted/No
PR	Q92K	26/1130	69/874	−1.86	<<2×10^−16^	<<0.001	B*15/Adapted/Yes
PR	L10I	345/1125	459/849	−1.41	<<2×10^−16^	<<0.001	B*15/Adapted/Yes
PR	I93L	417/1150	448/885	−0.85	<<2×10^−16^	<<0.001	B*15/Adapted/Yes
RT	Q207E	196/1169	216/880	−0.68	2×10^−5^	<0.001	B*15/Adapted/No
RT	Q207R	25/1169	8/880	1.25	0.03274	0.022	B*15/Adapted/Yes
RT	102R	56/1155	17/797	1.38	0.00041	<0.001	B*48/Adapted/Yes
RT	K103R	48/1127	9/835	2.03	2×10^−5^	<<0.001	B*48/Adapted/Yes
RT	R211G	43/1154	7/863	2.24	2×10^−5^	<<0.001	B*15/Adapted/No, A*32/Adapted/No
PR	L63S	66/1131	12/881	2.10	<<2×10^−16^	<<0.001	B*13/Adapted/Yes
PR	S162C	180/1209	94/867	2.10	<<2×10^−16^	<<0.001	B*07/Adapted/Yes
PR	L63S	66/1197	12/893	2.10	<<2×10^−16^	<<0.001	B*13/Adapted/Yes
PR	L63A	49/1197	11/893	1.77	<0.0001	<0.001	C*04/Adapted/Yes
PR	T12A	33/1197	6/898	2.07	0.0004	<<0.001	B*52/Adapted/Yes
PR	K14R	161/1165	21/893	1.10	<0.0026	<0.001	B*51/Adapted/Yes
PR	G16E	63/1187	14/853	1.77	<0.0001	<0.001	C*15/Adapted/Yes
PR	N37S	175/1200	53/879	1.71	<0.0001	<<0.001	B*44/Adapted/Yes
PR	P39S	37/1197	2/893	3.83	<<2×10^−16^	<<0.001	C*14/Adapted/Yes
RT	E203D	40/1145	70/867	−1.28	2×10^−5^	<<0.001	A*02/Non-adapted/No

A summarized result of amino acid residue frequencies that are significantly enriched with the absence of L90M mutation is shown in the table. The first column indicates what protein the mutation resides. The second column indicates the mutation and position of occurrence. The third and fourth column note the observed counts of a mutation in either the L90+ or M90+ set. Columns five to seven show the result of the fisher analysis and q-value calculation. The last column describe how the mutation is related to a specific HLA subtype. The first value is the HLA subtype in question, the second value is whether a mutation confers CTL escape, indicated as “ADAPTED” for escape and “NONADAPTED” for susceptibility. The last value indicate whether the mutation is directly involved in CTL escape or exist as a compensatory mutation. Values of deminished HLA subtype associated residues are included for reference purposes elsewhere in the article.

Referring back to Table 2, two substitutions, RT:Q207R (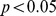, direct) and RT:R211G (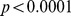, indirect) associated with HLA-B*15 were enriched. RT:R211G is also associated with A*32. The mutations RT:K102R and RT:K103R associated with HLA:B*48 mutation also occur at higher frequencies in the PR:90L set. Conflictingly, it appears that other mutations associated with HLA B*15 are found at lower frequencies. This is likely due the strong association of PR:90M with some substitutions that correlated with both drug resistance and HLA-B*15, namely PR:A71V and PR:L10I.

For subtype B*15, the residues in RT, 174H, 207H, 207R and 211G were used for assignment, while RT 102R and 103R were used for subtype B*48 assignment. A total of 120 patients (1929 sequences in total) were assigned the HLA:B*15 subtype and 118 patients (1938 sequences in total) the HLA B*48 subtype. The negative set which excluded both B*15+ and B*48+ assigned patients, consisted of 1799 patients (8469 sequences in total). HLA subtype A*32 was not included in subsequent frequency analysis, since the only available residue used for assignment, RT 211G both overlapped with HLA subtype B*15 and resulted in a too small set appropriate for statistical analysis, i.e. 32 patients.

The results in Table 3 demonstrate the significantly lower frequencies of L90M in sequences from patients with the assigned HLA-B*48 and HLA-B*15 subtypes. Significantly lower frequencies not only included L90M, but also major resistance mutations that tend to occur with PR 90 M. The frequencies commonly occurring PR mutations, M46I, I54V, A71V, V82A and I84V were also tested in both B*48 and B*15 subtype sequence sets. The extremely low q-values further support the calculated p-values. These results were a good indication that subtypes B*48 and B*15 possibly drive selection against HIV-1 protease sequences with a L90M substitution. However, due to the lower observed frequency of other PI resistance related mutations, it was further investigated whether the lower frequencies of L90M were truly due to the assigned HLA subtypes or due to a lack of acquired drug resistance.

**Table 3:** Frequency comparison of the L90M and correlated substitutions

Mutation	HLA Subtype	L90/ M90+	L90/ M90−	Odds	p-value	q-value
**L90M**	B*15	33/87	599/1221	0.52	0.001	0.006
	B*48	30/86	865/1008	0.41	<0.001	0.003
**M46I**	B*15	26/98	606/1210	0.53	0.004	0.011
	B*48	33/87	599/1221	0.76	0.230	0.256
**I54V**	B*15	22/102	690/1139	0.36	<<0.001	<0.001
	B*48	23/94	689/1147	0.41	<<0.001	<0.001
**A71V**	B*15	29/94	781/1049	0.42	<<0.001	0.003
	B*48	35/83	775/1060	0.58	0.007	0.015
**V82A**	B*15	30/92	619/1234	0.65	0.047	0.058
	B*48	23/96	626/1260	0.47	0.001	0.003
**I84V**	B*15	12/112	426/1444	0.36	<0.001	0.002
	B*48	12/106	426/1450	0.47	0.001	0.003

Results of the Fisher exact test of L90M, M46I, A71V, V82A and I84V between B*15+ or B*48+ sets and B*48−/B*15- sequence sets. The calculated odds ratios and associated p-values and q-values are shown. Most of the results show a significant p-values below 0.05 with q-values below 0.1, except for M46I in the B*48+ set which yielded a p-value and q-value of 0.230 and 0.256 respectively.

### The observed lower frequencies of other PI resistance mutations do not account for the lower observed frequencies of L90M

Factors that may influence the frequency of L90M are not necessarily limited to the presence of HLA subtypes B*15 or B*48. For instance, if the patients from the sequence B*48+ or B*15+ underwent a treatment plan that prefers Tipranavir (TPV) or Duranavir (DRV), lower frequencies could also be observed, since L90M does not factor in during development of resistance to these drugs [35]. The frequency differences of protease resistance mutations exclusive to TPV and DRV between B*48+ or B*15+ and B*15-/B*48- sequence sets were calculated. No significant differences of PR 82L (Tipranavir, major mutation) or PR 82F (DRV, minor mutation) were observed. Measurement of the impact of M46I, I54V, V71A, V82A, I84V frequencies on the frequency of L90M, was accomplished by the construction of a linear model. Five sequence sets were constructed, each devoid of M46I, I54V, V71A, V82A and I84V respectively, which were referred to as clean slate sets. A second five sequence set was constructed, each with sequences that contain one of the L90M correlated mutations, which were labeled the inclusion sets. The clean slate sets were then individually populated with an increasing amount of sequences from the inclusion set, ranging from 5% to 95%. At each step, resampling of was performed on both the inclusion set and the clean slates and the frequency of all the mutations, including L90M, were recorded. Ultimately, a total of 5000 frequency data points for each mutation were recorded for each set.

**Figure 2:**
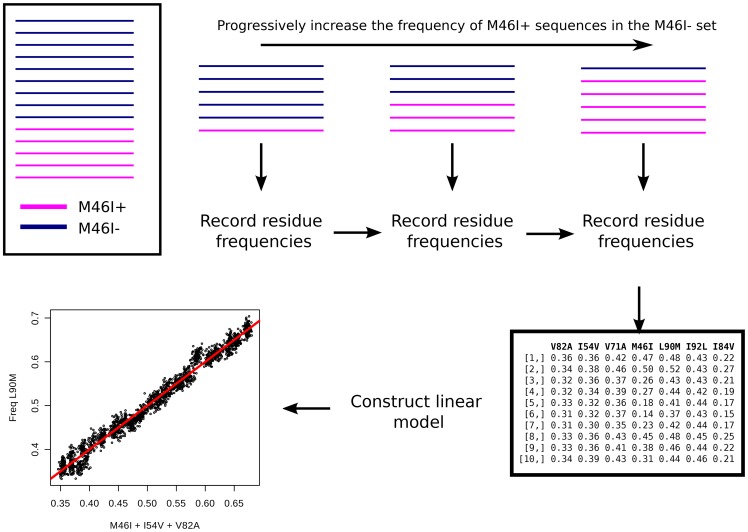
Construction of the L90M frequency linear model. The figure demonstrates the procedure to construct the data sets used to predict the expected frequency of L90M considering the frequency of co-occurring mutations. First, the sequence set is split into two groups; one devoid of the mutation of interest, in this case PR M46I, and the other that contains the mutation. Not that each line represents the entire sequence set obtained from a single patient. The patient ID in the mutation negative-set are increasingly replaced with patient sequence sets from the M46I group. At each step the frequencies for V82A, I54V, V71A, M46I, L90M and I84V were recorded. From this data, a linear model specific to a particular mutation, M46I in this case was constructed.

From this data, a linear model was constructed to predict expected frequencies of L90M in a sequence set by using the frequencies of L90M correlated mutations as input parameters. An overview of this process is demonstrated in Figure 2. A total of five linear models constructed that predict L90M frequencies, each tailored for a specific correlated mutation (please refer to the methods section). Instead of using a single parameter in each model to predict the frequency of L90M, each model was constructed using the frequencies of multiple L90M-correlated mutations. The rationale behind this approach was to avoid under-fitting due to a lack of parameters, and importantly, ensuring the model captures the variance of L90M in a random set. By referring to the HIVdb (http://hivdb.stanford.edu, [30], [36]), additional terms for the linear model were chosen based on observed PI resistance mutation patterns that occur in conjunction with the mutation and L90M. For instance, instead of measuring the singular effect of M46I frequency on L90M frequency, I84V was included as a second parameter in accordance with the relatively common M46I-I84V-L90M mutation pattern in PR sequences conferring high level resistance to nelfinavir (NFV), saquinavir (SQV), indinavir (IDV) and atazanavir (ATV). Common mutation patterns selected for each model were obtained from the HIVdb.

**Figure 3:**
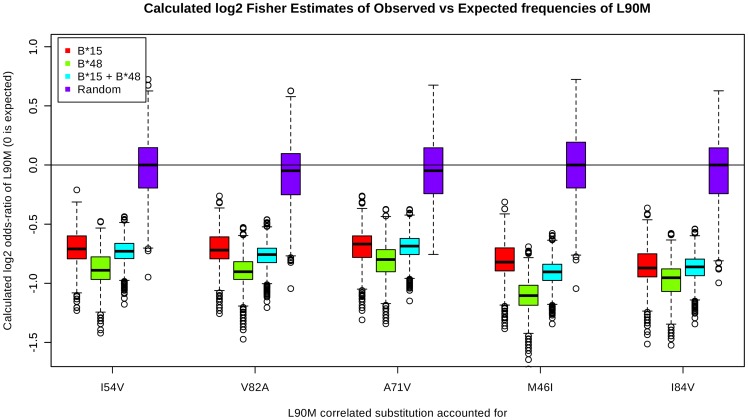
Box-and-whiskers plots of odds ratios observed between observed and expected frequencies of L90M produced by the linear models for various PI related mutations. The figure is a visual representation of the data listed in Table 4. The odds ratios were estimated from the result of the Fisher's exact test and converted to 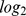 values for clarity purposes. The annotation of the x-axis tick marks represent the model used for calculation of the expected L90M frequency. The red, green, cyan and purple boxes each represent odds ratios calculated for B*15+, B*48+, B*15+/B*48+ and a random set. The random set is always centered around approximately zero while the rest of the box-and-whiskers plots are centered below −0.5. In all cases, the B*48 set shows the largest discrepancies to the random set.

The observed frequency of L90M in the B*48+ and B*15+ and B*48+/B*15+ combined sets were compared with the expected L90M frequency produced by the models by using a Fisher's exact test. This was done for the B*48+, B*15+ and a set including sequences from both B*48+ and B*15+ sets. Random sequence sets of size 120 were generated by sampling from PR sequences of 1799 patients. By resampling the sequence sets and re-applying the test, confidence intervals for the odds-ratios were calculated. The results are displayed in Figure 3 and Table 4. The tests clearly show a significant difference in frequency of the observed frequencies of L90M versus the expected frequencies produced by all the linear models. The robustness of the models are evident in that the expected L90M frequencies did not differ significantly for each tested mutation in all of the HLA subtype sets tested. By examining Figure 3, it became clear that the B*48 sets (green) were generally more significant than either the B*15+ (red) or B*15/B*48 (cyan) sets. The results are also significantly different than the results for the randomly selected sequence sets (purple), although a few outliers did overlap, which chance was attributed to. These results show that the lower observed frequencies of L90M in the B*15+ and B*48+ sets are not due to bias induced by antiretroviral choice.

**Table 4:** Frequency comparison of between the observed and correlated mutation sets for HLA B*15 and HLA B*48.

HLA Subtype	PR mutation	Sample	M90 (observed)	M90 (expected)	p-value	Odds-ratio (66.7% c.i.)
B*15	V82A	120	32/88	45/75	0.048	0.607 (0.557,0.664)
B*48	V82A	118	31/87	47/69	0.014	0.531 (0.481,0.583)
B*15/B*48	V82A	220	59/161	85/135	0.005	0.582 (0.554,0.626)
Random	V82A	120	53/67	53/67	0.500	0.967 (0.764,0.143)
B*15	I54V	120	32/88	45/75	0.048	0.607 (0.557,0.681)
B*48	I54V	118	31/87	47/71	0.019	0.540 (0.494,0.588)
B*15/B*48	I54V	220	59/161	84/136	0.007	0.459 (0.494,0.529)
Random	I54V	120	53/67	55/65	0.552	1.000(0.815,1.221)
B*15	V71A	120	32/88	45/75	0.048	0.607 (0.572,0.684)
B*48	V71A	118	31/87	46/72	0.026	0.559 (0.525,0.631)
B*15/B*48	V71A	220	59/161	83/137	0.002	0.583 (0.543,0.624)
Random	V71A	120	53/67	54/66	0.500	0.967 (0.812,1.181)
B*15	M46I	120	32/88	47/73	0.027	0.566 (0.519,0.637)
B*48	M46I	118	31/87	52/666	0.003	0.454 (0.425,0.512)
B*15/B*48	M46I	220	59/163	90/130	0.001	0.530 (0.499,0.572)
Random	M46I	120	53/67	53/67	0.602	1.034 (0.842,1.182)
B*15	I84V	120	32/88	52/78	0.020	0.547 (0.501,0.611)
B*48	I84V	118	31/87	48/70	0.010	0.503 (0.471,0.568)
B*15/B*48	I84V	220	59/161	89/131	0.002	0.540 (0.510,0.589)
Random	I84V	120	53/67	53/67	0.552	1.000 (0788,1181)

Results of the Fisher's exact test between observed and expected counts of L90M in sequence sets from patients annotated with B*15+, B*48+, B*15+ or B*48+, and a pool of random patients. The expected L90M counts for each associated PI resistance mutation were calculated with the linear models described elsewhere in this article. The 66.7% confidence intervals were calculated from a bootstrapped sets obtained from resampling the sequence sets and applied to the linear models. The observed L90M frequencies were significantly lower than the expected L90M frequencies.

### HLA subtypes other than HLA-B*15 and HLA-B*48 do not show significant negative correlation with L90M

It was relevant to this study to determine the influence of other HLA allotypes on L90M selection. Because of the HLA assignment strategy used here, i.e. using HLA-associated substitutions only in RT, not all HLA subtypes could be tested. HLA subtypes *excluded* were A*01, A*24, A*25, A*26, A*29, A*30, A*31, A*34, A*66, A*69, A*74, B*08, B*14, B*13, B*14, B*35, B*37, B*38, B*42, B*44, B*45, B*50, B*51, B*53, B*55, B*56, B*58, C*01, C*02, C*05, C*06, C*08, C*12, C*14, C*16, C*17 and C*18. HLA subtypes *included* were A*02, A*03, A*11, A*23, A*33, A*68, B*07, B*18, B*39, B*40, B*41, B*48. B*49, B*52, B*57, B*81, C*03, C*04 and C*07. In total, 6/21 (28%) of HLA-A subtypes, 11/29 (38%) of HLA-B subtypes and 3/13 (23%) of HLA-C subtypes were tested. The reason alternatives to RT (such as p17, p24, Int or Nef) were considered as sources of mutations to assign HLA allotypes, is due to the small amount of available HIV sequences that contain the PR L90M substitution in conjunction with p17, p24, Int or Nef sequences. The small sample yielded by inclusion these protein at HLA assignment rendered it inappropriate for robust analyses.

None of the tested HLA subtypes showed negative association with L90M, even after adjusting L90M frequencies with the linear models mentioned earlier. This included subtypes, which were shown earlier to have CTL escape residues associated with PR L90. Paradoxically, subtype C*03, which shows strong linkage disequilibrium with subtype B*15, was positively associated with L90M [37]. This result can be attributed to the mutation used for HLA assignment, i.e. RT substitution K43E. Although not listed as a mutation conferring drug resistance against RT inhibitors, this mutation was calculated to occur at a significantly higher frequency in sequences obtained from drug exposed patients versus sequences obtained from drug naïve patients (Odds-ratio 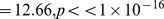). It was concluded that RT K43E could have caused false assignment of HLA subtype C*03 in Pol sequences obtained from drug exposed patients exhibiting other RT inhibitor resistance related mutations.

The assignment of HLA A*02 did not yield a significantly negative correlation with L90M result, despite L90M enhancing the PR 76–84 HLA-A*02 restricted epitope. A possible explanation is that the substitutions used for subtype A*02 assignment are not exclusive to A*02 and produced a significant proportion of false HLA assignments. To be thorough, A*02 was tested for negative correlation with an RT mutation that confers resistance against emtricitabine, abacavir and lamivudine, M184V. The mutation causes an increase in immunogenicity of an A*02 epitope spanning RT 181–189, YQYMDDLYV (Y9V) [38]. Sequence sets were divided based on the presence or absence of the mutation M184V, much like The sequence sets were divided based on the presence or absence of the M184V mutation. Because M184V is a reverse transcriptase inhibitor resistance mutation, only sequence with the common RTI mutation, L210W, were used in calculation to avoid bias sequences devoid of RT inhibitor resistance mutations. The results showed a decrease in the RT E203D mutation frequency (OR = 0.51, 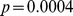) for the set that does not express the M184V mutation in RT. The E203D is flagged as NONADAPTED for the HLA*A*02 subtype and it can be assumed that lower occurrence of RT E203D is positively correlated with the presence of HLA*A*02. Although very indirect, this method could at least plausibly indicate lower frequencies of RT M184V in the presence of HLA*A*02. The conclusion is that assignment of HLA subtypes B*15 and B*48 using the selection strategy was not spurious.

### The L90M protease mutation increases predicted binding affinity of peptides to HLA allotypes belonging to subtypes A*32, B*15 and B*48

To find a possible causal relationship between the presence of HLA-B*48 or HLA-B*15 and the absence of L90M, IC50 values of peptides in the region 82-98 for representatives of various HLA subtypes were predicted with NetMHCPan. Significant results are listed in Table 5. The L90M substitution is predicted to have a significant positive effect on HLA binding for the HLA-B*15 (B*1503), HLA A*32 (A*3201) and HLA-B*48 (B*4802) subtypes, with only a marginal increase in predicted binding affinity for HLA-A*0201. The peptide, LMTQIGCTL (LM9L), produced NetCTLPan scores in the top 1% of the PR peptides for the allotypes B*1503 and B*4802. The peptide predicted to bind to subtype A*32, MTQIGCTLNF (M10F) was ranked among the 3%, providing weaker support of an HLA A*32 restricted epitope. NetChop predictions, which reveal potential proteasomal cleavage site, at the terminal end of the epitope (PR 97L). Proteasomal cleavage predictors are modest at best, but due to the high score predicted, i.e. 0.975 of a possible maximum score of 1.000, a false positive seemed unlikely. The C-terminal of the M10F peptide is already at a known cleavage site overlapping with T9F. To the author's knowledge, the peptides listed in Table 5 have not been shown to be epitopes anywhere in the literature, although L90M is known to enhance an HLA-A2 restricted epitope in the PR 76–84 region [18]. It is true that binding affinity does not necessarily constitute an HLA binder to be an epitope, but affinity does improve the stability of the peptide-HLA complex and increases the chance of contact with the appropriate CTL T-Cell Receptor [39], [40].

**Table 5:** Difference in NetMHCPan prediction scores between consensus PR peptides and residue substituted peptides.

Pos	Sub AA	Consensus	Substituted	HLA Allotype	Original Score	Substituted Score	*log_2_* IC50 change	Rank Cons/Rank Sub
82–90	L90M	VNIIGRNLL	VNIIGRNL M	B*1503	0.326	0.435	−1.70	Unknown/15.0
89–97	L90M	LLTQIGCTL	L M TQIGCTL	A*0201	0.462	0.499	−0.57	7.0/3.0
88–97	L90M	NLLTQIGCTL	NL M TQIGCTL	A*0201	0.467	0.556	−2.01	4.0/3.0
89–97	L90M	LLTQIGCTL	L M TQIGCTL	B*1503	0.569	0.749	−2.79	3.0/1.5
89–97	L90M	LLTQIGCTL	L M TQIGCTL	B*4802	0.486	0.701	−3.36	3.0/1.5
90–99	L90M	LTQIGCTLNF	M TQIGCTLNF	A*3201	0.373	0.534	−2.51	6.0/3.0

The reference name is in the format PROTEIN:HLA:EPITOPE. NetMHCPan scores are in 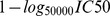 values. The IC50 change is the log value difference of the IC50 value in nM. Negative delta values indicate improved HLA binding. The last column show NetCTLPan rank results for the epitope before/after the L90M substitution.

It is worth mentioning that not the entire set of allotypes of subtypes B*15 and B*48 produced IC50 predicted values indicative of a significantly higher affinity. A total of 9/18 (50%) of HLA allotypes belonging to B*48 and 40/93 (43%) produced IC50 values below 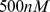 which is considered the threshold for sufficient binding affinity. This result indicate that not HLA allotypes belonging to subtypes may cause the induction of a CTL response against the putative epitopes.

### Mutations PR Q92K and Q92R are enriched in B*15+ and B*48+ sets

It was investigated whether other mutations within the PR 89–97 region were enriched in the B*48+ and B*15+ sequence sets. One mutation, PR I93L, is known to facilitate resistance to the HLA-B*15 restricted epitope spanning PR 90–99, TQIGCTLNF (T9F) [41]. The mutation Q92K is also associated with HLA escape referenced from the table used earlier. An additional mutation, Q92R was observed in the sequences. The mutation Q92R was found in addition to be enriched in both B*15+ (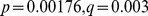) and B*48+ (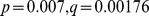) sequence sets. There was no evidence for enrichment of Q92K in the B*48+ sets.

Inferring a mechanism of escape cannot easily be accomplished computationally. Other researchers have shown in detail general mechanisms of escape that can include diminished interaction of a presented CTL epitope with a complementary cytotoxic T-cell receptor [13]. Another possible method of escape, is the induction of a highly active proteasomal cleavage site within the epitope [42]. The mechanism behind the CTL immunity escape of T9F provided by the PR I93L mutation is unknown. Indeed, it is shown that the CTL escape provided by the PR I93L has higher avidity in terms of ELISPOT analysis [17]. However, as shown in Table 6, I93L has a significant impact on proteasomal cleavage prediction score for position 93 in PR. This may cause the premature destruction of the epitope before it is loaded onto HLA class I. A significantly higher predicted proteasomal cleavage score for PR 92 was obtained by the Q92K substitution (0.549), but this score is very close to the threshold of 0.500 and could very well be a false positive. In conjunction with I93L, the proteasomal score was further increased to 0.735. However, the co-occurrence of Q92K and I93L is severely diminished in HIV PR sequences (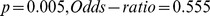). It seemed unlikely that another cleavage site would be necessary in close proximity of a highly probable proteasomal cleavage site. No significant diminished co-occurrence of Q92R and I93L was observed. Both the Q92K/R mutations did not have a profound negative impact on HLA binding affinity and did not change the rank percentage from the NetCTLPan results. It was therefore concluded a likely mechanism of escape would be through attenuated or abrogated interaction with a complementary cytotoxic T-cell receptor. This enrichment of substitutions internal to the predicted epitopes provide further evidence of a putative epitope in the region PR 89–97.

**Table 6:** NetChop3.0 results for the region spanning PR 88–99.

Position	Q92I93	Q92L93	K92I93	K92L93	R92I93	R92L93
89	0.406	**0.629**	0.423	**0.646**	0.454	**0.695**
90	0.240	0.404	0.252	0.427	0.343	**0.570**
91	0.036	0.045	0.031	0.036	0.031	0.037
92	0.062	0.090	**0.549**	**0.735**	0.251	0.374
93	**0.611**	**0.894**	**0.657**	**0.893**	**0.592**	**0.888**
94	0.115	0.148	0.074	0.094	0.057	0.071
95	0.030	0.030	0.026	0.026	0.026	0.026
96	0.083	0.059	0.087	0.062	0.081	0.059
97	**0.975**	**0.968**	**0.975**	**0.968**	**0.973**	**0.965**
98	0.218	0.219	0.247	0.249	0.228	0.229
99	**0.623**	**0.689**	**0.581**	**0.641**	**0.910**	**0.930**

The Q92I93 represents the results obtained from the consensus PR sequence of HIV-1 subtype B. Other columns show the scores obtained by substitutions at position 92 and 93. Using default parameters, a score above 0.500 indicate a predicted cleavage site.

## Discussion

The sequences from patients with the assigned HLA subtypes B*15, B*48 and A*32 demonstrated exclusive lower frequencies of L90M. Compensating for correlated mutations, whose lower frequency may bias the observed frequency of L90M, still yielded significant results. This lead to the conclusion that the diminished frequencies of L90M in the presence of HLA B*15 and B*48 are not spurious. Although the PR:76–84 HLA-A*02 restricted epitope could not be detected by analytical methods here, the enhanced Y9V epitope produced by RT:M184V was detected by observing higher frequencies of the HLA-A*02 negatively correlated RT mutation, E203D in the presence of RT M184V.

From the results, it is proposed that two novel epitopes LMTQIGCTL, HLA-B*15/B*48 (LM9L) and possibly MTQIGCTLF (M10F, HLA-A*32) are revealed by the PI resistance related mutation L90M and provide a causal relationship to decreased frequencies of L90M. The marked increase in affinity of the M9L to HLA subtypes B*15 and B*48 as well as a predicted increased affinity of M10F for A*32, provide a possible mechanism of epitope elucidation. However, the reason for subtype B*48 producing more significant results than B*15 remain unclear. It is possible that a greater range of HLA allotypes belonging to HLA B*48 can present LM9L than allotypes belonging to the subtype HLA B*15. Another reason could be false assignment of HLA B*15. False assignment would more likely render the HLA assigned sequence population homogenous to the general sequence population and render less significant result. False assignment can also happen due to the use CTL escape mutations retained in the HIV genome in a B*48−/B*15- host infected by HIV from a B*48+/B*15+ host. The T9F escape mutation, I93L, is predicted with high probability to produce a proteasomal cleavage site. This may also influence the amount of PR fragments available with intact, full length LM9L peptide. However, since the PR 89–99 region now contains a possible additional epitope, the higher probability of proteasomal cleavage may not be sufficient to provide complete escape. The fitness cost of PR Q92K/R is unknown and insufficient data was available here to do a rigorous analysis.

Further evidence are deduced from NetChop results that suggest a highly probable proteasomal cleavage site near the C-terminus of M9L, which is essential, since trimming of proteasomal fragments usually only occur at the N-terminus of the peptide. The predicted creation of a proteasomal cleavage site by Q92K, which is internal to both LM9L and M10F may be an escape mutation. Alternatively, when considering Q92R, it may attenuate interaction between the epitope and complementary cytotoxic T-cell receptor.

Even though the existence of LM9L and M10F may be proven with subsequent studies, there is no indication here of the immunogenicity of the epitope. The L90M mutation does appear to occur at lower frequencies, but the reason for the lack of complete elimination need to be investigated. Various issues, including, but not limited to, immunological hierarchies and false assignment of a sequence set to HLA subtypes B*15+ and B*48 could play a role. The contribution of L90M to viral fitness may also outweigh the immunological pressure induced by L90M.

Here no experimental evidence is supplied, but the multitude of statistical analysis and epitope prediction results do support the conclusion that there is potential for interaction between protease inhibitor resistance mutations and immunological selective pressure in hosts positive for HLA*B*15 and HLA-B*48 subtypes. Further research into validation of the results presented here are encouraged.

It should be emphasized that if either M9L or M10F is shown in subsequent studies to indeed be immunogenic CTL epitopes, it could be prudent to include PIs in treatment regiments such as Saquinavir or Nelfinavir for which L90M is a major resistance mutation. The novel epitopes could provide a protective effect and thus lower viral levels, delaying the progression of HIV infection to AIDS [20]. Even though only two overlapping potential epitopes are presented here, it is believed to be significant in supporting an argument that HLA allotype should be taken into consideration when patients start HIV treatment.

## Methods

### Obtaining HIV sequences

HIV amino acid sequences were obtained from the LANL HIV database (http://www.hiv.lanl.gov). All HIV protease (PR) and reverse transcriptase (RT) sequences of subtype B were obtained with annotations related to patient information including, where available, ARV exposure, HLA information and patient IDs. A total of 26,870 PR sequences from 10,049 patients and 25,342 RT sequences from 10,657 were used. Sequences from patients without patient ID annotations were discarded. Sequences that span the complete PR region (PR-RT) and first 217 amino acids of RT totaled 19,857 from 9,186 patients. For PR, 19% of patients were drug naïve, 25% were not drug naïve and the remainder were not annotated with treatment information. The RT and PR-RT sets showed similar values.

### Comparing residue frequencies between patients

A Fisher's exact test was performed to compare frequencies between groups of sequences. Sequences originating from the same patient were organized into sets using the Patient ID annotation as a reference. Residue counts were reduced to binary values, i.e. a residue was only counted once if it existed in the patient's sequence set. Residues matching the subtype B consensus sequence were only counted if there were no alternative residues in that position for a sequence set. Due to the binary count, there are incidences where the total sum for a sequence set may exceed the total number of patients within that set. For example, if 10 patients in the sequence set of 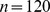 patients had sequences alternating between the PR residues PR 46I and PR46L, the total count would be 130. The effect of possible bias were measured to be marginal and mitigated by resampling procedures.

Whenever sequences were split according to criteria mentioned elsewhere in the article, sequences from patients that occur in both groups were excluded from analysis to avoid calculation errors and bias. Statistical analyses were performed using the R (www.r-project.org) and Python (http://www.python.org) programming languages [43]. Where appropriate, Storey's q-value was calculated to provide an estimation of the false discovery rate. Generally, p-values with an associated q-value of 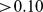 were regarded as non-significant.

### HLA assignment

Using the set of Karlsson *et al.* [18], HLA allotypes were assigned to patients based on the presence of mutations conferring CTL escape in any of their sequences. The dataset provides a q-value to assess the expected False Discovery Rate for each mutation. HLA associated mutations with a q-value of 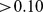 or those positively associated with drug resistance were excluded in HLA assignment.

### CTL epitope prediction

MHC affinity prediction was performed using a local copy of NetMHCPan 2.4 [25]. NetMHCPan produces a score from 0 to 1 and represents predicted 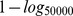 IC50 nM values of 8-11mer peptides for a vast range of HLA allotypes. Differences between peptides from the consensus HIV-1 Subtype B PR sequence and those produced by ARV resistance mutations were recorded. Significant results were considered to be those that cause a change 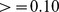 or changes that cross the binding threshold, which is set at 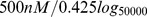 units. Further predictions were performed with NetCTLPan 1.2 server, which combine NetChop and NetMHCPan predictions and assigns a rank of the predicted MHC binder relative. The rank is the position of the score in a list of scores obtained from list of naturally occurring peptides. This is useful as an alternative to IC50 values especially in cases where prediction accuracy is low. Note that the current implementation of NetCTLPan uses an older version of NetMHCPan and the MHC binder results were updated to values produced by NetMHCPan 2.4. Where proteasomal cleavage prediction was exclusively needed, a local copy of NetChop 3.0 was used. The web version of POPISK was used for immunogenicity prediction.

### Linear model to predict expected L90M frequency

Linear models for the estimation of L90M frequency given a set of frequencies for co-occurring mutations were constructed. The influence of every mutation was measured individually. Sequence sets were divided into groups depending on the existence or absence of the mutation in question. The sequences in the negative set were increasingly replaced by sequences from the mutation positive set and frequencies of all the mutations recorded at each step. With each mutation, a data set with 5000 frequency data points for each mutation were recorded. For each mutation, other co-occurring mutations were included as additional terms for the equation. For each model, the frequency of L90M, i.e. P(L90M) was calculated by a linear addition of the intercept together with the values produced by the other terms. The terms were chosen by observing frequently occurring combinations of PI resistance mutations listed in the hivDB together with L90M. Evaluation revealed the models were good general predictors of L90M substitution frequency. Equations 1, 2, 3, 4 and 5 represent L90M frequency estimators trained from the V82A, I54V, A71V, M46I and I84V sets respectively. Where 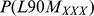 is the expected frequency of L90M and 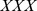 denote the mutation under which the model was trained. The 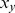 variables correspond to the frequency of the mutation denoted by the 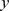 subscript. The 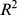 values are the calulated adjusted 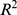 values for the model.
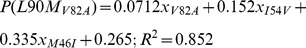
(1)

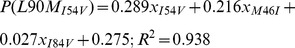
(2)

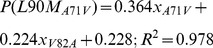
(3)

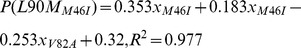
(4)

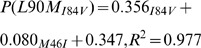
(5)


## References

[1] Weiss RA (1993) How does HIV cause AIDS? Science 260: 1273–1279. 8493571 10.1126/science.8493571

[2] Borrow P, Lewicki H, Hahn BH, Shaw GM, Oldstone MB (1994) Virus-specific CD8+ cytotoxic T-lymphocyte activity associated with control of viremia in primary human immunodeficiency virus type 1 infection. J Virol 68: 6103–6110. 8057491 10.1128/jvi.68.9.6103-6110.1994 PMC237022

[3] York IA, Rock KL (1996) Antigen processing and presentation by the class i major histocompatibility complex. Annu Rev Immunol 14: 369–396. 8717519 10.1146/annurev.immunol.14.1.369

[4] Sidney J, Peters B, Frahm N, Brander C, Sette A (2008) HLA class I supertypes: a revised and updated classification. BMC Immunol 9: 1. 18211710 10.1186/1471-2172-9-1 PMC2245908

[5] Migueles SA, Sabbaghian MS, Shupert WL, Bettinotti MP, Marincola FM, et al (2000) HLA B*5701 is highly associated with restriction of virus replication in a subgroup of HIV-infected long term nonprogressors. Proc Natl Acad Sci U S A 97: 2709–2714. 10694578 10.1073/pnas.050567397 PMC15994

[6] Kaslow RA, Rivers C, Tang J, Bender TJ, Goepfert PA, et al (2001) Polymorphisms in HLA class I genes associated with both favorable prognosis of human immunodeficiency virus (HIV) type 1 infection and positive cytotoxic T-lymphocyte responses to ALVAC-HIV recombinant canarypox vaccines. J Virol 75: 8681–8689. 11507213 10.1128/JVI.75.18.8681-8689.2001 PMC115113

[7] Altfeld M, Addo MM, Rosenberg ES, Hecht FM, Lee PK, et al (2003) Inuence of HLA-B57 on clinical presentation and viral control during acute HIV-1 infection. AIDS 17: 2581–2591. 14685052 10.1097/00002030-200312050-00005

[8] Pereyra F, Addo MM, Kaufmann DE, Liu Y, Miura T, et al (2008) Genetic and immunologic heterogeneity among persons who control HIV infection in the absence of therapy. J Infect Dis 197: 563–571. 18275276 10.1086/526786

[9] Roberts JD, Bebenek K, Kunkel TA (1988) The accuracy of reverse transcriptase from HIV-1. Science 242: 1171–1173. 2460925 10.1126/science.2460925

[10] Preston BD, Poiesz BJ, Loeb LA (1988) Fidelity of HIV-1 reverse transcriptase. Science 242: 1168–1171. 2460924 10.1126/science.2460924

[11] Wood N, Bhattacharya T, Keele BF, Giorgi E, Liu M, et al (2009) HIV evolution in early infection: selection pressures, patterns of insertion and deletion, and the impact of APOBEC. PLoS Pathog 5: e1000414. 19424423 10.1371/journal.ppat.1000414 PMC2671846

[12] Yokomaku Y, Miura H, Tomiyama H, Kawana Tachikawa A, Takiguchi M, et al (2004) Impaired processing and presentation of cytotoxic-T-lymphocyte (CTL) epitopes are major escape mechanisms from CTL immune pressure in human immunodeficiency virus type 1 infection. J Virol 78: 1324–1332. 14722287 10.1128/JVI.78.3.1324-1332.2004 PMC321367

[13] Iversen AKN, Stewart Jones G, Learn GH, Christie N, Sylvester Hviid C, et al (2006) Conicting selective forces affect T cell receptor contacts in an immunodominant human immunodeficiency virus epitope. Nat Immunol 7: 179–189. 16388312 10.1038/ni1298

[14] Patel K, Hernn MA, Williams PL, Seeger JD, McIntosh K, et al (2008) Long-term effectiveness of highly active antiretroviral therapy on the survival of children and adolescents with HIV infection: a 10-year follow-up study. Clin Infect Dis 46: 507–515. 18199042 10.1086/526524

[15] Menndez-Arias L (2002) Targeting hiv: antiretroviral therapy and development of drug resistance. Trends Pharmacol Sci 23: 381–388. 12377580 10.1016/s0165-6147(02)02054-0

[16] Mason RD, Bowmer MI, Howley CM, Gallant M, Myers JCE, et al (2004) Antiretroviral drug resistance mutations sustain or enhance CTL recognition of common HIV-1 Pol epitopes. J Immunol 172: 7212–7219. 15153547 10.4049/jimmunol.172.11.7212

[17] Mueller SM, Schaetz B, Eismann K, Bergmann S, Bauerle M, et al (2007) Dual selection pressure by drugs and HLA class I-restricted immune responses on human immunodeficiency virus type 1 protease. J Virol 81: 2887–2898. 17202219 10.1128/JVI.01547-06 PMC1866003

[18] Karlsson AC, Chapman JM, Heiken BD, Hoh R, Kallas EG, et al (2007) Antiretroviral drug therapy alters the profile of human immunodeficiency virus type 1-specific T-cell responses and shifts the immunodominant cytotoxic T-lymphocyte response from Gag to Pol. J Virol 81: 11543–11548. 17670829 10.1128/JVI.00779-07 PMC2045537

[19] Mueller SM, Spriewald BM, Bergmann S, Eismann K, Leykauf M, et al (2011) Inuence of major 19 HIV-1 protease inhibitor resistance mutations on CTL recognition. J Acquir Immune Defic Syndr 56: 109–117. 21107269 10.1097/QAI.0b013e3181fe946e

[20] Stratov I, Dale CJ, Chea S, McCluskey J, Kent SJ (2005) Induction of T-cell immunity to antiretroviral drug-resistant human immunodeficiency virus type 1. J Virol 79: 7728–7737. 15919925 10.1128/JVI.79.12.7728-7737.2005 PMC1143690

[21] Petrovsky N, Brusic V (2002) Computational immunology: The coming of age. Immunol Cell Biol 80: 248–254. 12067412 10.1046/j.1440-1711.2002.01093.x

[22] De Groot AS (2006) Immunomics: discovering new targets for vaccines and therapeutics. Drug Discov Today 11: 203–209. 16580597 10.1016/S1359-6446(05)03720-7

[23] Tenzer S, Peters B, Bulik S, Schoor O, Lemmel C, et al (2005) Modeling the MHC class I pathway by combining predictions of proteasomal cleavage, TAP transport and MHC class I binding. Cell Mol Life Sci 62: 1025–1037. 15868101 10.1007/s00018-005-4528-2 PMC11924537

[24] Nielsen M, Lundegaard C, Lund O, Kemir C (2005) The role of the proteasome in generating cytotoxic T-cell epitopes: insights obtained from improved predictions of proteasomal cleavage. Immunogenetics 57: 33–41. 15744535 10.1007/s00251-005-0781-7

[25] Nielsen M, Lundegaard C, Blicher T, Lamberth K, Harndahl M, et al (2007) NetMHCpan, a method for quantitative predictions of peptide binding to any HLA-A and -B locus protein of known sequence. PLoS One 2: e796. 17726526 10.1371/journal.pone.0000796 PMC1949492

[26] Tung CW, Ho SY (2007) POPI: predicting immunogenicity of MHC class I binding peptides by mining informative physicochemical properties. Bioinformatics 23: 942–949. 17384427 10.1093/bioinformatics/btm061

[27] Tung CW, Ziehm M, Kmper A, Kohlbacher O, Ho SY (2011) POPISK: T-cell reactivity prediction using support vector machines and string kernels. BMC Bioinformatics 12: 446. 22085524 10.1186/1471-2105-12-446 PMC3228774

[28] Stranzl T, Larsen MV, Lundegaard C, Nielsen M (2010) NetCTLpan: pan-specific MHC class I pathway epitope predictions. Immunogenetics 62: 357–368. 20379710 10.1007/s00251-010-0441-4 PMC2875469

[29] Brumme ZL, John M, Carlson JM, Brumme CJ, Chan D, et al (2009) HLA-associated immune escape pathways in HIV-1 subtype B Gag, Pol and Nef proteins. PLoS One 4: e6687. 19690614 10.1371/journal.pone.0006687 PMC2723923

[30] Shafer RW (2006) Rationale and uses of a public HIV drug-resistance database. J Infect Dis 194 Suppl 1S51–S58. 16921473 10.1086/505356 PMC2614864

[31] Marchand C, Maddali K, Mtifiot M, Pommier Y (2009) HIV-1 IN inhibitors: 2010 update and perspectives. Curr Top Med Chem 9: 1016–1037. 19747122 10.2174/156802609789630910 PMC2860603

[32] Di Giambenedetto S, Prosperi M, Fanti I, Bruzzone B, Paolucci S, et al (2011) Update on emergence of HIV-1 resistance to antiretroviral drug classes in an Italian national database: 2007–2009. Clin Microbiol Infect 17: 1352–1355. 21635664 10.1111/j.1469-0691.2011.03563.x

[33] Johnson VA, Calvez V, Gnthard HF, Paredes R, Pillay D, et al (2011) 2011 update of the drug resistance mutations in HIV-1. Top Antivir Med 19: 156–164. 22156218 PMC6148877

[34] Storey JD (2002) A direct approach to false discovery rates. Journal of the Royal Statistical Society: Series B (Statistical Methodology) 64: 479–498.

[35] Johnson VA, Calvez V, Gunthard H, Paredes R, Pillay D, et al (2013) Update of the drug resistance mutations in hiv-1: March 2013. Top Antivir Med 21: 4–12. PMC6148891 23596273

[36] Rhee SY, Gonzales MJ, Kantor R, Betts BJ, Ravela J, et al (2003) Human immunodeficiency virus reverse transcriptase and protease sequence database. Nucleic Acids Res 31: 298–303. 12520007 10.1093/nar/gkg100 PMC165547

[37] Layrisse Z, Fernandez M, Montagnani S, Matos M, Balbas O, et al (2000) Hla-c* 03 is a risk factor for cardiomyopathy in chagas disease. Human immunology 61: 925–929. 11053636 10.1016/s0198-8859(00)00161-0

[38] Vollbrecht T, Eberle J, Roider J, Bhler S, Stirner R, et al (2012) Control of M184V HIV-1 mutants by CD8 T-cell responses. Med Microbiol Immunol 201: 201–211. 22200907 10.1007/s00430-011-0222-1

[39] Pogue RR, Eron J, Frelinger JA, Matsui M (1995) Amino-terminal alteration of the HLA-A*0201-restricted human immunodeficiency virus pol peptide increases complex stability and in vitro immunogenicity. Proc Natl Acad Sci U S A 92: 8166–8170. 7545295 10.1073/pnas.92.18.8166 PMC41117

[40] Sette A, Vitiello A, Reherman B, Fowler P, Nayersina R, et al (1994) The relationship between class I binding affinity and immunogenicity of potential cytotoxic T cell epitopes. J Immunol 153: 5586–5592. 7527444

[41] Frahm N, Kiepiela P, Adams S, Linde CH, Hewitt HS, et al (2006) Control of human immunodeficiency virus replication by cytotoxic T lymphocytes targeting subdominant epitopes. Nat Immunol 7: 173–178. 16369537 10.1038/ni1281

[42] Cardinaud S, Consiglieri G, Bouziat R, Urrutia A, Graff-Dubois S, et al (2011) CTL escape mediated by proteasomal destruction of an HIV-1 cryptic epitope. PLoS Pathog 7: e1002049. 21589903 10.1371/journal.ppat.1002049 PMC3093368

[43] R Core Team (2012) R: A Language and Environment for Statistical Computing. R Foundation for Statistical Computing, Vienna, Austria. URL http://www.R-project.org/. ISBN 3-900051-07-0.

